# The Pharmacological Profile of Second Generation Pyrovalerone Cathinones and Related Cathinone Derivative

**DOI:** 10.3390/ijms22158277

**Published:** 2021-07-31

**Authors:** Karolina E. Kolaczynska, Jan Thomann, Marius C. Hoener, Matthias E. Liechti

**Affiliations:** 1 Division of Psychopharmacology Research, Department of Biomedicine, University Hospital Basel and University of Basel, 4031 Basel, Switzerland; karolina.kolaczynska@unibas.ch (K.E.K.); jan.thomann@unibas.ch (J.T.); 2 Neuroscience Research, pRED, Roche Innovation Center Basel, F. Hoffmann-La Roche Ltd., 4070 Basel, Switzerland; marius.hoener@roche.com

**Keywords:** novel psychoactive substance, pyrovalerone, cathinone, monoamine, transporter, receptor, inhibition

## Abstract

Pyrovalerone cathinones are potent psychoactive substances that possess a pyrrolidine moiety. Pyrovalerone-type novel psychoactive substances (NPS) are continuously detected but their pharmacology and toxicology are largely unknown. We assessed several pyrovalerone and related cathinone derivatives at the human norepinephrine (NET), dopamine (DAT), and serotonin (SERT) uptake transporters using HEK293 cells overexpressing each respective transporter. We examined the transporter-mediated monoamine efflux in preloaded cells. The receptor binding and activation potency was also assessed at the 5-HT_1A_, 5-HT_2A_, 5-HT_2B_, and 5-HT_2C_ receptors. All pyrovalerone cathinones were potent DAT (IC_50_ = 0.02–8.7 μM) and NET inhibitors (IC_50_ = 0.03–4.6 μM), and exhibited no SERT activity at concentrations < 10 μM. None of the compounds induced monoamine efflux. NEH was a potent DAT/NET inhibitor (IC_50_ = 0.17–0.18 μM). 4F-PBP and NEH exhibited a high selectivity for the DAT (DAT/SERT ratio = 264–356). Extension of the alkyl chain enhanced NET and DAT inhibition potency, while presence of a 3,4-methylenedioxy moiety increased SERT inhibition potency. Most compounds did not exhibit any relevant activity at other monoamine receptors. In conclusion, 4F-PBP and NEH were selective DAT/NET inhibitors indicating that these substances likely produce strong psychostimulant effects and have a high abuse liability.

## 1. Introduction

Synthetic cathinones are a large subgroup of designer drugs, commonly known as “legal highs” or “bath salts”, which mimic the psychostimulant effects of classical drugs of abuse, including 3,4-methylenedioxymethamphetamine [MDMA], amphetamine or cocaine [1,2,3]. Sold often on the Internet at low-cost, with labels such as “not for human consumption”, these novel psychoactive substances are synthesized as legal alternatives to classical drugs of abuse, thereby bypassing drug control legislation, and often marketed as safer alternatives [4,5]. However, these substances rarely have well-defined pharmacological and toxicological profiles and often pose a huge burden on public health due to their associated adverse effects and potential toxicities. Common adverse effects include tachycardia, hallucinations, agitation, and violent outbursts, all of which can lead to life-threatening situations [6,7,8].

Structurally analogous to cathinone, the naturally occurring psychoactive agent is found in the khat plant (*Catha edulis*); this subgroup of designer drugs consists of several members possessing different structural moieties at either the phenyl ring, central carbon, or at the nitrogen atom [2,9,10]. A subset includes the pyrovalerone cathinones, which bare a pyrrolidine ring at the nitrogen atom e.g., α-pyrrolidinopropiophenone (α-PPP) [2,9,10]. Interestingly, pyrovalerone cathinones stand out among other cathinones in their pharmacological effects, as they mimic the psychostimulant effects of amphetamines as well as behave as pure transporter blockers of the monoamine uptake transporters [9,11,12,13].

3,4-methylenedioxypyrovalerone (MDPV), a key member of the pyrovalerone cathinones subgroup, is the most frequently detected and abused cathinone found in “legal highs” preparations in Europe and the US [6,14,15,16]. As a highly potent inhibitor of the dopamine (DAT) and norepinephrine (NET) transporters but not serotonin transporter (SERT), MDPV produces sympathomimetic and psychostimulant effects in users and is associated with a high risk of abuse, similar to amphetamine or cocaine [13,17,18]. Structurally similar derivatives of MDPV are continuously synthesized in order to overcome the regulation imposed on MDPV and its related cathinones [19,20,21]. The production of second generation pyrovalerone cathinones like α-pyrrolidinopentiophenone (α-PVP), α-pyrrolidinobutiophenone (α-PBP), 3,4-methylenedioxy-α-pyrrolidinopropiophenone (MDPPP), and 3,4-methylenedioxy-α-pyrrolidinobutiophenone (MDPBP) poses a problem for the regulators as these novel substances are found in “legal high” preparations and require regulation. Moreover, they are also problematic to users as they have not been properly investigated for their pharmacological and toxicological properties [21,22,23,24]. It is therefore essential to generate pharmacological data for new substances in order to predict their in vivo effects [25], and their potential adverse side effects as some of them may be linked to severe toxicological events [26,27].

**Figure 1:**
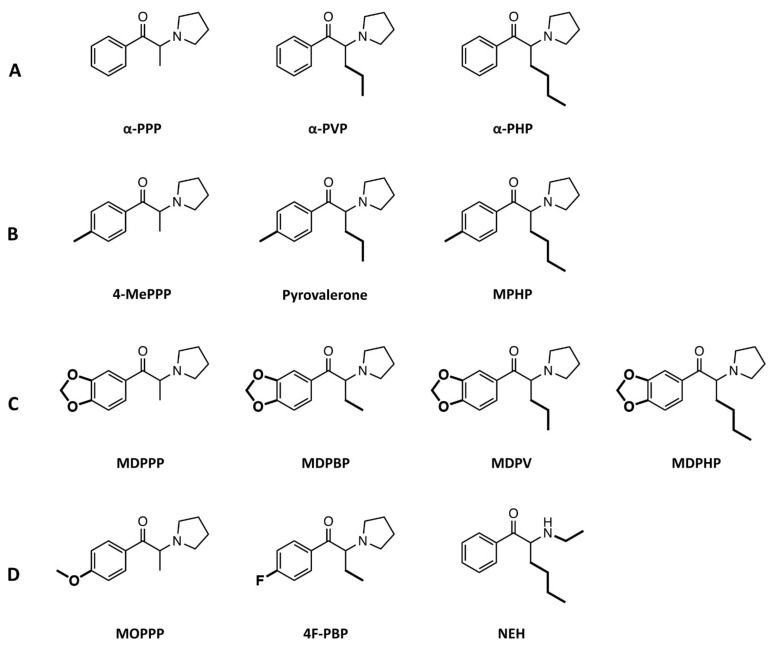
Chemical structures of pyrovalerone cathinones and NEH. Substances subdivided into (**A**) compounds with substitutions only onto the α-carbon either methyl (α-PPP), propyl (α-PVP) or butyl group (α-PHP), (**B**) compounds with a 4-methyl group and an insertion of a methyl (4-MePPP), propyl (pyrovalerone) or butyl group (MPHP) on to the α-carbon group, (**C**) compounds with 3,4-methylenedioxy insertion onto ring structure (MDPPP) and insertion of ethyl (MDPBP), propyl (MDPV) or butyl (MDPHP) group onto the α-carbon moiety, and (**D**) compounds with a 4-methoxy (MOPPP) or 4-fluorine substitution on ring structure, and related cathinone lacking the pyrrolidine moiety at the nitrogen atom (NEH). All described structural modifications are indicated in bold.

Our group has previously investigated the in vitro pharmacology of some pyrovalerone cathinones, including MDPV, MDPPP, MDPBP, naphyrone, pyrovalerone, and α-PVP [13]. All of these compounds potently inhibited the NET and DAT but not SERT with the exception of naphyrone, which also inhibited the SERT. Moreover, none of the pyrovalerones produce any monoamine efflux, in contrast to ring-substituted cathinones like mephedrone [13,18]. Due to the influx of more second generation pyrovalerone cathinones appearing in “bath salt” preparations [19,20,21,28], we wanted to investigate the in vitro pharmacological profiles of new derivatives to see how they compare to previously described pyrovalerone cathinone members (Figure 1). Although pharmacological data exists for some of these investigated derivatives (Marusich et al. 2014, Eshleman et al. 2017), we aim to confirm these results using our assay set up and additionally provide data for the substances that have been unexplored thus far using the same assay used to characterize first generation pyrovalerones and many other NPS [13,18,25]. Overall, we examined whether each compound inhibited the reuptake of the three monoamines at the NET, DAT, and SERT, additionally exploring the potential of each substance to induce transporter-mediated release of the monoamines. Furthermore, we investigated the receptor binding and activation properties of these pyrovalerone cathinones at the human serotonergic (5-HT_1A_, 5-HT_2A_, 5-HT_2B_, and 5-HT_2C_) receptors. 

For the purposes of providing a full pharmacological characterization of each compound at the monoamine transporters and receptors, including dopaminergic, adrenergic receptors, and the trace-amine associated receptor 1 (TAAR1), we also compiled data from previous publications by our group [13,18,29,30].

## 2. Results

### 2.1. Monoamine Uptake Transporter Inhibition

**Figure 2:**
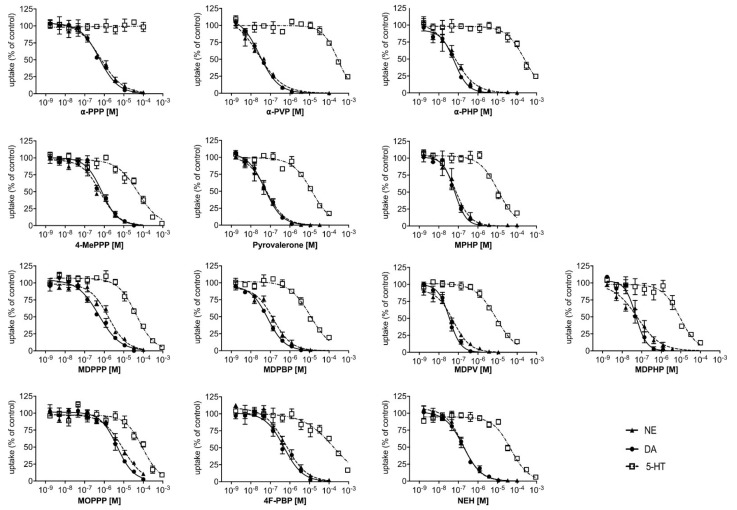
Monoamine uptake inhibition of pyrovalerone cathinones and NEH. Monoamine uptake inhibition of norepinephrine (NE), dopamine (DA), or serotonin (5−HT) were assessed in human embryonic kidney 293 (HEK293) cells stably transfected with each corresponding uptake transporter (NET, DAT, or SERT, respectively). Curves were fitted by non-linear regression and data is shown as mean ± standard error mean (SEM) with corresponding IC_50_ values for each compound presented in Table 1.

The monoamine uptake inhibition curves at the human NET, DAT, and SERT are illustrated in Figure 2. Corresponding inhibition potencies (IC_50_) of the pyrovalerone cathinones at each respective transporter are shown in Table 1. 

The pyrovalerone cathinones with progressively longer carbon chain at the α-carbon group (Figure 1A; α-PPP, α-PVP, and α-PHP, respectively) were potent inhibitors at the NET and DAT (IC_50_ = 0.02 − 0.64 μM), but exhibited little activity at the SERT (Figure 2, Table 1). High DAT/SERT inhibition ratios (> 1000) meant these compounds were the most selective inhibitors of the dopaminergic transporter in the entire series. Progressive extension of the α-carbon group (α-PPP, methyl → α-PVP, propyl → α-PHP, butyl) enhanced the inhibition potency at the NET and DAT.

Similarly, the pyrovalerones with 4-methyl moiety and extensions of the α-carbon group (Figure 1B; 4-MePPP, pyrovalerone, and MPHP, respectively) were highly selective inhibitors of DAT (DAT/SERT inhibition ratios > 72) and NET (IC_50_ = 0.06 − 0.64 μM), and exhibited an increased potency at the SERT (IC_50_ = 11 − 55 μM). Similarly, the progressive extension of the α-carbon methyl group increased the IC_50_ at the DAT and NET, and also at the SERT.

The pyrovalerone containing the 3,4-methylenedioxy moiety and different extensions of the α-carbon group (Figure 1C; MDPPP, MDPBP, MDPV, and MDPHP, respectively) interacted with the NET and DAT at submicromolar concentrations (IC_50_ < 1 μM; Table 1), with the exception of MDPPP which inhibited the NET at micromolar concentration (IC_50_ = 1.7 μM). The DAT/SERT inhibition ratios exhibited by these substances reflected the high selectivity for the dopamine transporter (DAT/SERT ratio = 80–241). Overall, the 3,4-methylenedioxy moiety enhanced the inhibition potency of these compounds to the SERT in comparison to their counterparts (mainly α-PPP, α-PVP, and α-PHP lacking the moiety), although the SERT inhibition potency was still relatively low compared to IC_50_ observed at DAT or NET.

Finally, 4-methoxy containing MOPPP interacted with the NET and DAT at micromolar concentrations (IC_50_ = 8.7 μM and 4.6 μM, respectively; Figure 1D), exhibiting the least potent interactions at the transporters out of all examined substances in the entire series. With low inhibition potency at the SERT (IC_50_ > 10 μM), MOPPP reflects a greater selectivity for the DAT vs. SERT. The 4-fluorinated analog of α-PBP, 4F-PBP potently inhibited the NET and DAT (IC_50_ < 0.61 μM; Table 1), but showed littler potency at the SERT (IC_50_ > 100 μM), similar to the profiles observed for the pyrovalerone cathinones with progressively longer chain at the α-carbon methyl group. Likewise, NEH was a potent inhibitor at the NET and DAT (IC_50_ < 0.18 μM; Table 1) with a selectivity for the DAT vs. SERT inhibition ratio of 264, similar to that of MDPV.

### 2.2. Serotonergic Receptor Interactions: Binding Affinity and Activation Potency

The serotonin receptor binding affinities and activation potencies for the human 5-HT_1A_, 5-HT_2A_, 5-HT_2B_, and 5-HT_2C_ receptors are presented in Table 2. All but two of the pyrovalerone cathinones bound to the h5-HT_1A_ receptor in the micromolar range (*K_i_* ≤ 13 μM) similar to binding affinity observed for MDMA and amphetamine (*K_i_* = 6.7–11 μM). MOPPP and NEH were the only cathinones that did not bind to the receptor in the examined concentration range (*K_i_* ≥ 17 μM). In contrast, most of the compounds did not bind to the h5-HT_2A_ receptor (*K_i_* ≥ 13 μM), whereas α-PPP, 4-MePPP, MPHP, and MDPPP exhibited low micromolar binding to the receptor (*K_i_* = 1.1–8.0 μM) similar to MDMA (*K_i_* = 6.3 μM). None of the pyrovalerone cathinones interacted with the h5-HT_2C_ receptors in the examined concentration range (*K_i_* ≥ 5.1 μM). Overall, all of the compounds in the series did not exhibit any relevant activation potency at either the 5-HT_2A_ or 5-HT_2B_ receptors (EC_50_ ≥ 10 μM).

### 2.3. Monoamine Transporter and Non-Serotonergic Receptor Binding Interactions

The binding affinity at the monoamine transporters and at the monoaminergic receptors are presented in Table 3. The examined pyrovalerone cathinones bound to the NET (*K_i_* = 0.06–3.5 μM) and DAT (*K_i_* = 0.007–0.18 μM) with high affinity, in line with their high inhibition potency at these two transporters. Furthermore, some compounds, mainly pyrovalerone, MDPPP, MDPBP, MDPHP, and MDPV exhibited relevant affinity at the SERT albeit in the low micromolar range (*K_i_* = 2.9–12 μM).

Overall, the pyrovalerone cathinones did not interact at any relevant concentrations with the dopaminergic D_2_ receptor, adrenergic α_1A_ and α_2A_ receptors or trace-amine associated receptor 1 (TAAR1; human, rat, and mouse), with the exception of 4-MePPP, which bound the adrenergic α_1A_ receptor in the low micromolar range (*K_i_* = 2.2 μM).

### 2.4. Transporter-Mediated Monoamine Efflux

**Figure 3:**
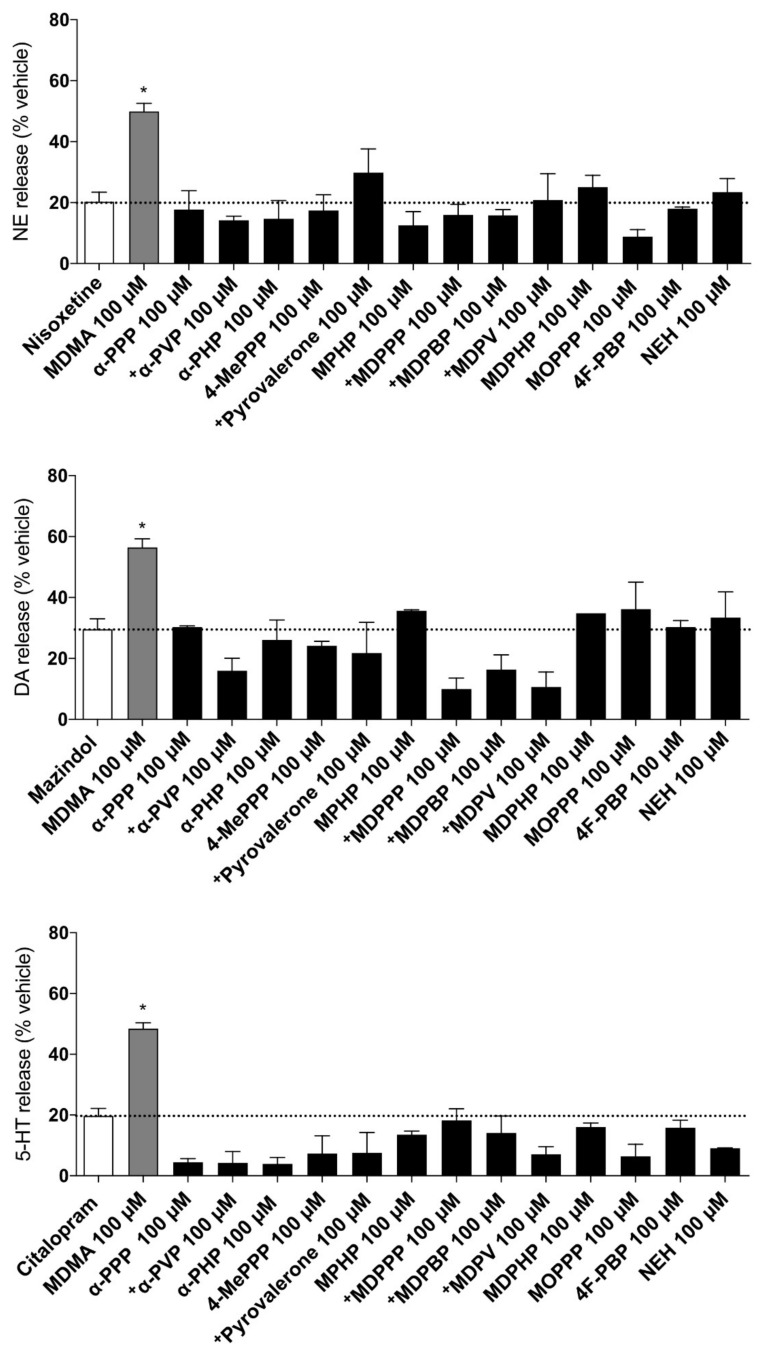
Monoamine efflux induced by 100 μM of drug in HEK293 cells expressing human NET, DAT, or SERT preloaded with radiolabeled monoamine. Each compound’s induced monoamine efflux was measured as a percentage of a respective radiolabelled neurotransmitter ([^3^H]-NE/DA/5-HT) decrease in the monoamine preloaded cells compared to the control. Nonspecific “pseudo-efflux” that occurs as a result of monoamines diffusion and subsequent reuptake inhibition has been indicated as a dashed line on the graphs. A substance was a monoamine transporter substrate if it produced significantly (* *p* < 0.05) more monoamine efflux than each respective pure uptake inhibitor (nisoxetine, mazindol, or citalopram). Data presented is shown as the mean ± SEM. Monoamine efflux data for some pyrovalerone cathinones (indicated as ^+^) was adapted from a previous publication [13].

The transporter-mediated monoamine efflux at 100 μM of each test drug are shown in Figure 3. MDMA was used as a positive control as it causes significant release at the transporters when compared to each respective inhibitor. Overall, none of the examined pyrovalerone cathinones induced significant release of the monoamines at the examined concentration, indicating that all of the compounds act as pure uptake blockers at the transporters.

## 3. Discussion

### 3.1. Monoamine Transporter Inhibition and Transporter-Mediated Efflux

The simplest pyrovalerones of the series, mainly α-PPP, α-PVP, and α-PHP (Figure 1A) all interacted with the monoamine transporters in a similar manner, mainly potently inhibiting the NET and DAT, with little activity at the SERT (Figure 2, Table 1). α-PVP and α-PHP exhibited a 32-fold and 11-fold higher inhibition potency at the NET, respectively, when compared to α-PPP. Likewise, at the DAT, 17-fold and 9-fold higher potency was observed for α-PVP and α-PHP, indicating that the extension of the alkyl chain length caused progressively more potent inhibition at the NET and DAT. This finding is consistent with previous studies by [11,12], which support our findings in both rat brain synaptosomes and transfected HEK293 cells. Furthermore, all three pyrovalerones were highly selective for the DAT vs. SERT, which is associated with a higher abuse potential [31,32]. In vivo studies extend these findings in the discriminative stimulus effect paradigm in rats, where these pyrovalerones are substituted for classical drugs of abuse, such as cocaine and methamphetamine [33]. Moreover, all three pyrovalerones did not induce any monoamine efflux in preloaded cells (Figure 3), confirming their status as pure transporter uptake blockers [11].

The pyrovalerones with a 4-methyl moiety (4-MePPP) and/or extensions of the α-carbon group (pyrovalerone and MPHP) were also potent inhibitors at the transporters with 4-MePPP exhibiting an 11-fold and 15-fold lower potency at the NET and DAT, respectively when compared to pyrovalerone and MPHP (Figure 1B and Figure 2, Table 1) [11,34,35]. Interestingly, all three pyrovalerones exhibited a higher potency at the SERT compared to the simplest pyrovalerones of the series (α-PPP, α-PVP, and α-PHP). They were, however, potent DAT inhibitors (DAT/SERT ratio = 72–169) much in line with previously reported DAT/SERT ratios by [11,34]. The presence of the 4-methyl moiety had little effect on the inhibition potency observed at the NET and DAT [11,34]. Similar to the simplest pyrovalerones of the series, both MPHP and 4-MePPP were pure transporter uptake blockers, as no monoamine efflux was observed (Figure 3). In vivo studies examining 4-MePPP’s neurochemical and behavioural effects suggest that the substance is likely to be self-administered as it causes a dose-dependent surge in dopamine levels in the nucleus accumbens and initiates forward locomotion and stereotypical movements in rodents [35,36]. However, in comparison to highly dopaminergic pyrovalerones like α-PPP, 4-MePPP is likely to have a lower abuse potential as it is less dopaminergic (lower DAT/SERT ratio) and recent in vivo studies have shown that it does not induce conditioned place preference in rats, and can only be fully substituted for methamphetamine but not cocaine [37].

The 3,4-methylenedioxy containing pyrovalerones (Figure 1C) with various extension of the α-carbon group were pure and potent inhibitors at the NET and DAT (Figure 3) [11,34] and exhibited similar inhibition potency at the SERT as observed by pyrovalerones described above (Figure 1B). Interestingly, MDPPP exhibited the lowest inhibition potency at the NET and DAT when compared to the remaining 3,4-methylenedioxy containing pyrovalerones, which in turn inhibited both transporters but with higher inhibition potency (8-fold to 34-fold higher). Furthermore, extension of the α-carbon group also enhanced the inhibition potency at the SERT, exhibited by a progressively lower IC_50_ value when comparing MDPPP, MDPBP, MDPV, and MDPHP (in such order), all in line with previous studies by [11,34]. Overall, the presence of the 3,4-methylenedioxy group merely enhanced SERT inhibition potency [11,34], as indicated by the higher IC_50_ values observed for MDPPP/MDPV/MDPHP when compared to their 3,4-methylenedioxy lacking counterparts, α-PPP, α-PVP, and α-PHP (Table 1). 

MOPPP, which possess a 4-methoxy moiety on the phenyl ring (Figure 1D), exhibited the lowest inhibition potency at the NET out of all investigated compounds, with an IC_50_ value about 14-fold higher than observed for α-PPP (Table 1). A similar observation could be made at the DAT where MOPPP displayed 8-fold lower inhibition potency with a IC_50_ value of 4.6 μM, when compared to α-PPP which bares no modification at the 4′-position. Moreover, MOPPP inhibited the SERT but to a far lesser extent and displayed the lowest DAT vs. SERT ratio out of all investigated compounds. To our best knowledge, MOPPP has not been previously examined at the monoamine uptake transporters. Our findings suggest that it does not inhibit the monoamine uptake transporters as potently as other herein investigated substances e.g., α-PPP. With a selectivity for the DAT vs. SERT, it is likely to induce psychostimulant-like effects and be associated with a high abuse liability [31]. MOPPP has previously detected in contaminated synthetic cathinone mixtures [38] and so far was mainly studied for its metabolism and toxicological screening [39,40].

On the other hand, 4F-PBP which contains a 4-fluorine moiety on the phenyl ring (Figure 1D), potently inhibited the NET and DAT, with a profile similar to α-PPP. Like all herein investigated pyrovalerones, 4F-PBP did not induce monoamine efflux (Figure 3), and was a potent DAT vs. SERT pure blocker, with a DAT/SERT ratio similar to pyrovalerone and MDPV. Furthermore, this suggests that the addition of the fluorine moiety onto the 4-position at the phenyl ring does not augment the inhibition profile of 4F-PBP’s fluorine-free counterpart, α -PBP as it seems to be also be very potent at the DAT and NET, but not SERT, as reported by [11]. Similar to MOPPP, 4F-PBP has been found in seized cathinone products [41] but has not been previously investigated at the monoamine transporters. Our findings suggest that with 4F-PBP’s potent inhibition profile with a strong selectivity at the DAT vs. SERT, it is likely to exhibit potent psychostimulant effects in users (similar to that of α-PPP) and be associated with a high abuse liability [31], similar to substances with more pronounced effects at the DAT vs. SERT. 

NEH, a synthetic cathinone lacking a pyrrolidine ring, also potently inhibited the NET and DAT, while exhibiting a much lower potency at the SERT (Table 1). Moreover, NEH did not produce any monoamine efflux (Figure 3), confirming its role as a pure and potent monoamine blocker at the NET and DAT. NEH’s strong inhibition selectivity for the DAT vs. SERT, indicates that the substance will produce mainly psychostimulant effects and be associated with a high abuse liability similar to pyrovalerone or MDPV. These findings are in line with previous reports of NEH’s effect at the monoamine uptake transporters [34]. Similar to other cathinones, NEH has been detected in synthetic cathinone products from as early as 2017 [42,43]. Recent reports of NEH’s associated fatal toxicity [42] and its recommended scheduling by the World Health Organization in 2020 [44], indicate that the substance is likely to be abused.

### 3.2. Serotonergic Receptor Binding and Activation Interactions 

Overall, the pyrovalerone cathinones interacted mostly with the 5-HT_1A_ and 5-HT_2A_ receptors. α-PVP, MDPV, MDPBP, and 4F-PBP bound to the 5-HT_1A_ receptor in a similar range to amphetamine (*K_i_* ≤ 10 μM). Meanwhile, all remaining compounds with the exception of NEH and MOPPP, bound to the receptor with less affinity similar to MDMA (*K_i_* ≥ 10 μM). Interestingly, most compounds did not interact with the 5-HT_2A_ receptor which is the responsible target site for the associated psychedelic effects induced by classical psychedelic substances like LSD or psilocybin [45,46,47,48]. Only α-PPP, 4-MePPP, MPHP, and MDPPP bound to the 5-HT_2A_ receptor in the low micromolar range (*K_i_* ≤ 10 μM) similar to the prototypical entactogen MDMA, which also produces psychedelic-like effects in users [49,50]. Previously, binding affinity at the receptor has been a useful predictor of the clinical dose needed to produce psychedelics effects by various stimulants [25]. None of the four pyrovalerones however activated the 5-HT_2A_ receptor, therefore it is unlikely that these substances will produce any relevant psychedelic-like effects in vivo, however this requires further investigations to be confirmed. Moreover, no relevant binding was observed for any of the examined cathinones at the corresponding 5-HT_2C_ receptor, which is also involved but to a much lesser extent, in the overall psychological profile produced by psychedelics [46]. The activation of the 5-HT_2B_ receptor was assessed in order to assess the potential of drug-induced endocardial fibrosis associated with the receptor [51,52]. None of the substances activated the 5-HT_2B_ receptor in the examined concentration range, indicating that they are unlikely to be associated with this type of cardiotoxicity.

### 3.3. Monoamine Transporter and Non-Serotonergic Receptor Binding Interactions

The pyrovalerones mainly interacted with the NET and DAT, binding to the transporters with high affinity ranging from 0.06 to 3.5 μM. At the SERT, most pyrovalerones exhibited a much lower binding affinity, which taken all together supported the monoamine uptake inhibition profiles of the substances and confirmed their action as inhibitors and not substrates at the transporters [11,13,18]. No relevant interactions were observed at the D_2_ dopaminergic receptor, the human TAAR1 or the mouse TAAR1 [13,18]. Likewise, no pyrovalerone bound to the α_1A_ adrenergic receptor, with the exception of 4-MePPP. The α_1A_ adrenergic receptor has been previously linked to physiological process such as vasoconstriction and hyperthermia, which commonly produced in users taking stimulants [53]. In contrast, 4-MePPP and other pyrovalerones did not interact with the α_2A_ adrenergic receptor in the examined concentration range, with the exception of MDPPP and MDPBP, which bound the receptor in the low micromolar range. Previously, the α_2A_ adrenergic receptor has been reported in its control of norepinephrine release and sympathomimetic toxicity [54]. Only α-PVP, MDPPP, and MDPV bound the rat TAAR1, an important receptor involved in the auto inhibition of stimulant effects induced by amphetamines in rodents [13,18,55]. 

## 4. Materials and Methods

### 4.1. Compounds

The investigated drugs, including 3,4-methylenedioxymethamphetamine (MDMA), 3,4-methylenedioxpyrovalerone (MDPV), 3,4-methylendioxy-α-pyrrolidinobutiophenone (MDPBP), pyrovalerone, α-pyrrolidinopropiophenone (α-PPP), and α-pyrrolidinopentiophenone (α-PVP) were purchased from Lipomed (Arlesheim, Switzerland), while α-pyrrolidinohexanophenone (α-PHP), 4-fluoro-α-pyrrolidinobutiophenone (4-fluoro-α-PBP), N-ethylamine-hexanophenone (NEH), 3,4-methylenedioxy-α-pyrrolidinopropiophenone (MDPPP), 4-methoxy-α-pyrrolidinopropiophenone (MOPPP), 3,4-methylenedioxy-α-pyrrolidinohexanophenone (MDPHP), and 4-methyl-α-pyrrolidinohexanophenone (MPHP) were purchased from Adipogen AG (Liestal, Switzerland). Finally, 4-methyl-α-pyrrolidinopropiophenone (4-MePPP) was synthesized by ReseaChem GmbH (Burgdorf, Switzerland). All aforementioned substances were in hydrochloride form with a purity of >98.5%. 

Radiolabelled monoamine neurotransmitters [^3^H]-norepinephrine (NE; 10.0 Ci/mmol) and [^3^H]-dopamine (DA; 45.4 Ci/mmol) were purchased from Perkin–Elmer (Schwerzenbach, Switzerland), while [^3^H]-serotonin (5-HT; 80.0 Ci/mmol) was obtained from Anawa (Zurich, Switzerland). The monoamine selective inhibitors for the dopamine and serotonin transporters, mazindol and fluoxetine, respectively, were purchased from Lipomed (Arlesheim, Switzerland), while the selective norepinephrine inhibitor, nisoxetine, was obtained from Sigma-Aldrich (Buchs, Switzerland).

### 4.2. Monoamine Uptake Transporter Inhibition

The monoamine uptake transporter inhibition was examined in accordance with previously described methods by [56,57] for each substance of interest.

Human embryonic kidney 293 (HEK293) cells (Invitrogen, Zug, Switzerland) stably transfected with the human norepinephrine (hNET), dopamine (hDAT), or serotonin (hSERT) uptake transporters were briefly cultured in Dulbecco’s modified Eagle’s medium (DMEM; Gibco, Life Technologies, Zug, Switzerland) containing 10% fetal bovine serum and 250 μg/mL geneticin (Gibco, Life Technologies, Zug, Switzerland). Next, the cells were detached at a confluency of 70−90% and resuspended in Krebs–Ringer Bicarbonate Buffer (Sigma-Aldrich, Buchs, Switzerland) at a concentration of 3 × 10^6^ cells per ml. The uptake buffer was additionally supplemented with 0.2 mg/mL of ascorbic acid (Sigma–Aldrich, Buchs, Switzerland) for the [^3^H]-DA uptake experiments.

In summary, using round bottom 96-well plates, 100 μL of cell suspension was incubated in 25 μL of buffer containing test substances, vehicle control (0.7% dimethyl sulfoxide, DMSO), or 10 μM of the respective monoamine uptake inhibitor, mainly fluoxetine (SERT), nisoxetine (NET), or mazindol (DAT) for 10 min at room temperature while on a rotary shaker at 450 rpm (Thermomixer Comfort, Eppendorf, Hamburg, Germany). Thereafter, the monoamine uptake transporter was initiated by the addition of 50 μL of each respective radiolabelled neurotransmitter ([^3^H]-5-HT, [^3^H]-DA or [^3^H]-NE) dissolved in the uptake buffer to a final concentration of 5 nM for an additional 10 min. Then, 100 μL of cell suspension mixture was transferred to microcentrifuge tubes containing 50 μL of 3 M potassium hydroxide (KOH, Sigma–Aldrich, Buchs, Switzerland) and 200 μL silicon oil (1:1 mixture of silicon oil type AR20 and AR200; Sigma–Aldrich, Buchs, Switzerland). Immediately after, the tubes were centrifugated (3 min, 13200 rpm) to terminate the uptake reaction by allowing the cells to move through the silicon oil into the KOH, which then lysed the cells. Quickly after, the tubes were immediately frozen with liquid nitrogen. Afterwards, the frozen cell pellet was cut off into 6 mL scintillation vials (Perkin–Elmer, Schwerzenbach, Switzerland) filled with 500 μL of lysis buffer (5 mM EDTA, 0.05 M TRIS-HCl, 50 mM NaCl and 1% NP-40 in water). Directly after the vials were shaken for 1 h at 700 rpm and then each vial was filled with 3 mL of scintillation fluid (Ultimagold, Perkin–Elmer, Schwerzenbach, Switzerland). The uptake of the monoamines was measured using a liquid scintillation counter (Packard Tri-Carb Liquid Scintillation Counter 1900 TR). Specific monoamine uptake was examined by subtracting the nonspecific uptake in the presence of selective inhibitor from the total counts measured.

The data were analysed using Prism software (version 8, GraphPad, San Diego, CA, USA) and was fitted by a nonlinear regression to variable-slope sigmoidal dose-response curve. The IC_50_ values were extracted in order to determine each drug’s inhibition potency at the various monoamine transporters. Furthermore, the DAT/SERT ratio expressed as 1/DAT IC_50_: 1/SERT IC_50_ was calculated to assess whether a substance exhibited stronger serotonergic effects (ratio < 1, more entactogenic and similar to MDMA) or stronger dopaminergic effects (ratio > 1, more psychostimulant and similar to cocaine).

### 4.3. Monoamine Efflux Mediated by the Transporters 

Monoamine efflux was assessed using human embryonic kidney 293 (HEK293) cells (Invitrogen, Zug, Switzerland) stably transfected with the human norepinephrine (hNET), dopamine (hDAT), or serotonin (hSERT) uptake transporters. In summary, the cells were seeded in poly-D-lysine coated XF24 cell culture microplates (Seahorse Biosciences, North Billerica, MA, USA) and cultured overnight at a concentration of 100,000 cells per well. 

The cells were then exposed to Krebs–HEPES release buffer (85 μL/well) comprised of 130 mM NaCl, 1.3 mM KCl, 2.2 mM CaCl_2_, 1.2 mM MgSO_4_, 1.2 mM KH_2_PO_4_, 10 mM HEPES, and 10 mM D-glucose at a pH of 7.5, which also contained 10 nM radiolabelled neurotransmitter ([^3^H]-5-HT for SERT, [^3^H]-DA for DAT or [^3^H]-NE for NET), 1 μM unlabelled neurotransmitter (DA or NE, only), 10 μM pargyline, and 0.2 mg/mL of ascorbic acid. This enabled the loading of the cells with their respective neurotransmitters via the uptake transporters. Afterwards, the release buffer was replaced by fresh buffer to wash the cells twice and the cells were then incubated for 15 min (DAT and SERT) or 45 min (NET) to 100 μM of test drugs dissolved in 1 mL of Krebs–HEPES buffer while shaking at 300 rpm and 37 °C. Termination of the release reaction occurred by removing the buffer from the cells and washing them with ice-cold buffer. Next, 50 μL of lysis buffer was added to the cells for 1 h in order to lyse the cells. Thereafter, 40 μL of the lysed cell mixture was transferred into scintillation vials (Perkin–Elmer, Schwerzenbach, Switzerland) containing 3 mL of scintillation fluid (Ultimagold, Perkin–Elmer, Schwerzenbach, Switzerland) and measured using liquid scintillation (as previously described in Section 2.2).

To quantify the “pseudo efflux” caused by the non-transporter mediated monoamine release and successive reuptake inhibition [58], each monoamine efflux experiment included a control where the cells were exposed to each respective transporter blocker (nisoxetine for NET, mazindol for DAT, and citalopram for SERT). The radioactivity inside the cells without any drug was set as 100%. The nonspecific release was subtracted from the total observed release at 100 μM of each examined drug to calculate the specific transporter mediated release. One single concentration of the test drugs was used (100 μM) and release exposure durations were set based on previously evaluated kinetics of the release-over-time curves [56].

A substance which produced significantly (*p*-value < 0.05) higher monoamine efflux compared to the efflux observed in the presence of a monoamine transporter inhibitor, was identified as a monoamine releaser. An ANOVA followed by a Holm–Sidak test was conducted to compare each drug’s specific transporter-mediated efflux based on at least three independent experiments to the control condition.

### 4.4. Radioligand Binding, Activation Potency, and Efficacy at the 5-HT Receptors

The radioligand binding assays for the human 5-HT_1A_, 5-HT_2A_, and 5-HT_2C_ receptors were examined as previously described by [59]. In summary, the human embryonic kidney 293 (HEK293) cell preparations were stably transfected with the human 5-HT_1A_, 5-HT_2A_ or 5-HT_2C_ receptors and then incubated with their respective radiolabelled ligands at a concentration equal to the dissociation constant, *K_d_*. The following radioligands were used for the 5-HT_1A_, 5-HT_2A_, and 5-HT_2C_ receptors, respectively; 0.90 nM [^3^H]8-hydroxy-2-(dipropylamino) tetralin (8-OH-DPAT), 0.40 nM [^3^H]ketanserin, and 1.4 nM [^3^H]mesulgerine. The radioligand displacement for each examined drug was determined. Specific binding of the radioligand to the target was calculated by subtracting the nonspecific binding (in the presence of the receptor’s competitor in excess) from the total observed binding. The following radioligand competitors were used for the 5-HT_1A_, 5-HT_2A_, and 5-HT_2C_ receptors, respectively; 10 μM pindolol (5-HT_1A_ receptor), 10 μM spiperone (5-HT_2A_ receptor), and 10 μM mianserin (5-HT_2C_ receptor).

The activation potency and efficacy of the 5-HT_2A_ and 5-HT_2B_ receptors were assessed as previously described by [59]. Mouse embryonic fibroblasts (NIH-3T3) were transfected with the human 5-HT_2A_ receptors, seed into 96-well plates coated with poly-D-lysine at a concentration of 70,000 cell per 0.1 mL and then incubated for 1 h at 37 °C in HEPES-Hank’s Balanced Salt Solution (HBSS) buffer (Gibco). Next, the plates were exposed to 100 μL/well of dye solution for 1 h at 37 °C inside the FLIPR (fluorescence imagining plate reader [FLIPR] calcium 5 assay kit; Molecular Devices, Sunnyvale, CA, USA). Then, the plates were exposed to the 25 μL/well of test drugs dissolved in HEPES–HBSS buffer and 250 mM probencid during online mode. The corresponding concentration vs. response curves were fitted using nonlinear regression and the EC_50_ values were calculated. The efficacy (maximal activity) was calculated relative to 5-HT activity, which was defined as 100%.

Human embryonic kidney 293 (HEK293) cells were transfected with the human 5-HT_2B_ receptors, seed into 96-well plates coated with poly-D-lysine at a concentration of 50,000 cell per 0.1 mL and then incubated for overnight at 37 °C in high glucose Dulbecco’s modified Eagle’s medium (DMEM; Invitrogen, Zug, Switzerland) containing 10% fetal calf serum (non-dialyzed, heat-inactivated), 250 mg/L Geneticin, and 10 mL/L PenStrep (Gibco). The medium was then removed using snap inversion and the cells were incubated with the 100 μL/well of calcium indicator Fluo-3-solution for 45 min at 31 °C (Molecular Probes, Eugene, OR, USA). Thereafter, the solution was removed using snap inversion and replaced with fresh calcium indicator Fluo-3-solution for an additional 45 min at 31 °C. The cells were then washed with HBSS and 20 mM HEPES using the EMBLA cell washer and immediately after incubated with 100 μL of assay buffer. The plate was then put into the FLIPR, turned online and the test drugs, diluted in the assay buffer were added to the corresponding wells (25 μL/well). The corresponding concentration vs. response curves were fitted using nonlinear regression and the EC_50_ values were calculated. The efficacy (maximal activity) was calculated relative to 5-HT activity, which was defined as 100%.

## 5. Conclusions

In the present study, we investigated the in vitro pharmacological profiles of several first and second generation pyrovalerone cathinones with various structural modifications. Overall, we observed that all of the compounds were potent inhibitors of norepinephrine and dopamine uptake transporters, and to far lesser extent at the serotonin uptake transporter. 4F-PBP and NEH strongly inhibited the DAT vs. SERT and did not induce any monoamine efflux. Both substances had comparable DAT/SERT ratio profiles to that of MDPV or pyrovalerone, indicating that both substances are likely to produce strong psychostimulant effects in users and have an associated high abuse potential.

Finally, most of the compounds interacted with moderate to low affinity at the 5-HT_1A_ receptor, while at all remaining receptors (5-HT_2A_, 5-HT_2B_, 5-HT_2C_ receptors, dopaminergic D_2_ receptor, adrenergic receptors, and human, rat, or mouse TAAR1) no relevant activity was observed for most compounds.

## Figures and Tables

**Table 1:** Monoamine transporter inhibition.

	NET	DAT	SERT	DAT/SERT Ratio
	IC_50_ [μM] (95% CI)	IC_50_ [μM] (95% CI)	IC_50_ [μM] (95% CI)	Ratio (95% CI)
***Pyrovalerone cathinones***				
α-PPP	0.64 (0.41–0.99)	0.56 (0.40–0.76)	>1000	>1000
α-PVP	0.02 (0.01–0.05)	0.03 (0.02–0.04)	279 (209–372)	>1000
α-PHP	0.06 (0.03–0.12)	0.06 (0.05–0.08)	245 (173–348)	>1000
4-MePPP	0.64 (0.43–0.95)	0.75 (0.58–0.97)	55 (38–78)	72 (39–134)
Pyrovalerone	0.06 (0.04–0.09)	0.05 (0.04–0.08)	13 (10–18)	256 (125–450)
MPHP	0.07 (0.05–0.10)	0.06 (0.05–0.08)	11 (8.0–15)	169 (100–300)
MDPPP	1.7 (1.3–2.2)	0.54 (0.37–0.79)	43 (34–55)	80 (43–149)
MDPBP	0.14 (0.10–0.19)	0.07 (0.06–0.09)	11 (8.6–15)	155 (96–250)
MDPV	0.05 (0.04–0.08)	0.03 (0.03–0.05)	8.4 (6.6–11)	241 (132–367)
MDPHP	0.06 (0.03–0.13)	0.05 (0.04–0.07)	9 (6.0–14)	184 (86–350)
MOPPP	8.7 (6.3–12)	4.6 (3.4–6.3)	94 (65–135)	20 (4.0–40)
4F-PBP	0.61 (0.43–0.87)	0.50 (0.36–0.69)	177 (97–325)	356 (141–903)
***Other***				
NEH	0.17 (0.12–0.24)	0.18 (0.14–0.24)	47 (38–60)	264 (158–429)
***Reference substances***				
MDMA	0.41 (0.33–0.52)	13 (11–16)	1.6 (1.2–2.2)	0.12 (0.08–0.20)
Amphetamine	0.07 (0.05–0.1) ^a^	1.3 (0.8–2.0) ^a^	45 (24–85) ^a^	35 (12–106) ^a^

Values are mean and 95% confidence intervals (CI). DAT/SERT ratio = (1/DAT IC_50_):(1/SERT IC_50_). Data previously published in ^a^ Rickli et al. (2015).

**Table 2:** Serotonin receptor binding affinities and activation potencies of pyrovalerone and NEH.

	h5-HT_1A_	h5-HT_2A_		h5-HT_2B_	h5-HT_2C_
	Receptor Binding	Receptor Binding	Activation Potency	Activation Potency	Receptor Binding
	*K*_i_ ± SD [μM]	*K*_i_ ± SD [μM]	EC_50_ ± SD [μM]	EC_50_ ± SD [μM]	*K*_i_ ± SD [μM]
	[^3^H]-8-OH-DPAT	[^3^H]-Ketanserin			[^3^H]-Mesulgerine
***Pyrovalerone cathinones***					
α-PPP	0.7 ± 0.3	1.1 ± 0.3	>20	>20	>15
α-PVP	6.1 ± 0.7	>13	>20	>20	>15
α-PHP	11 ± 5.3	>13	>20	>20	>15
4-MePPP	12 ± 2.2	1.3 ± 0.4	>10	>10	>5.1
Pyrovalerone	13 ± 1.4	>13	>20	>20	>15
MPHP	13 ± 1.9	7.3 ± 1.2	>20	>20	>15
MDPPP	1.9 ± 0.8	8.0 ± 1.2	>20	>20	>15
MDPBP	9.0 ± 1.5	>13	>20	>20	>15
MDPV	7.7 ± 0.6	>13	>20	>20	>15
MDPHP	13 ± 1.9	>13	>20	>20	>15
MOPPP	>17	>13	>20	>20	>15
4F-PBP	6.0 ± 3.4	>13	>20	>20	>15
***Other***					
NEH	>17	>13	>20	>20	>15
***Reference substances***					
MDMA	11 ± 2.0 ^a^	6.3 ± 2.4 ^b^	6.1 ± 0.3 ^b^	>20 ^b^	4.4 ± 0.8 ^a^
Amphetamine	6.7 ± 1.4 ^c^	>13 ^c^	NA	9.4 ± 1.6 ^b^	>13 ^c^

*K_i_* and EC_50_ values are given as mean ± SD; NA, not assessed. Data previously published in ^a^ Luethi, Kolaczynska et al. (2019), ^b^ Rickli et al. (2015), and ^c^ Simmler et al. (2013).

**Table 3:** Monoamine transporter and receptor binding affinities of pyrovalerone and NEH.

	hNET	hDAT	hSERT	D_2_	α_1A_	α_2A_	Human TAAR1	Rat TAAR1	Mouse TAAR1
	Receptor Binding						Activation Potency	Receptor Binding	
	*K*_i_ ± SD [μM]						EC_50_ ± SD [μM]	*K*_i_ ± SD [μM]	
	N-Methyl-[^3^H]-nisoxetine	[^3^H]-WIN-35,428	[^3^H]-Citalopram	[^3^H]-Spiperone	[^3^H]-Prazosin	[^3^H]-Rauwolscine		[^3^H]-RO5166017	
***Pyrovalerone cathinones***									
α-PPP	NA	NA	NA	NA	NA	NA	NA	NA	NA
α-PVP	0.06 ± 0.02 ^d^	0.007 ± 0.002 ^d^	>30 ^d^	>10 ^d^	>15 ^d^	>20 ^d^	NA	16 ± 6.4 ^d^	>20 ^d^
α-PHP	NA	NA	NA	NA	NA	NA	NA	NA	NA
4-MePPP	2.5 ± 0.7	0.3 ± 0.02	>7.4	>13	2.2 ± 0.1	>4.7	NA	NA	NA
Pyrovalerone	0.06 ± 0.01 ^c^	0.03 ± 0.01 ^c^	5.0 ± 0.3 ^c^	>30 ^c^	>6 ^c^	>20 ^c^	NA	>12.5 ^c^	>10 ^c^
MPHP	NA	NA	NA	NA	NA	NA	NA	NA	NA
MDPPP	3.5 ± 1.0 ^d^	0.18 ± 0.05 ^d^	12 ± 1.0 ^d^	>10 ^d^	>15 ^d^	14 ± 0.9 ^d^	NA	16 ± 6.7 ^d^	>20 ^d^
MDPBP	1.1 ± 0.1 ^d^	0.02 ± 0.002 ^d^	4.1 ± 1.2 ^d^	>20 ^d^	>4.9 ^d^	9.4 ± 1.6 ^d^	NA	>20 ^d^	>20 ^d^
MDPV	0.08 ± 0.02 ^c^	0.01 ± 0.002 ^c^	2.9 ± 0.1 ^c^	>30 ^c^	>6 ^c^	>20 ^c^	>30 ^b^	7.2 ± 1.1 ^c^	>10 ^c^
MDPHP	NA	NA	NA	NA	NA	NA	NA	NA	NA
MOPPP	NA	NA	NA	NA	NA	NA	NA	NA	NA
4F-PBP	NA	NA	NA	NA	NA	NA	NA	NA	NA
***Other***									
NEH	NA	NA	NA	NA	NA	NA	NA	NA	NA
***Reference substances***									
MDMA	>8.7 ^a^	>8.5 ^a^	>7.5 ^a^	>13 ^a^	6.9 ± 1.2 ^a^	4.6 ± 1.1 ^a^	35 ± 21 ^b^	0.25 ± 0.01 ^a^	3.1 ± 0.7 ^a^
Amphetamine	1.0 ± 0.6 ^c^	5.7 ± 3.8 ^c^	>25 ^c^	>30 ^c^	>6.0 ^c^	2.8 ± 0.8 ^c^	2.8 ± 0.8 ^b^	0.23 ± 0.18 ^c^	0.09 ± 0.06 ^c^

*K_i_* and EC_50_ values are given as mean ± SD; NA, not assessed. Data adapted from the following publications and used for comparison: ^a^ Luethi, Kolaczynska et al. (2019), ^b^ Simmler et al. (2016), ^c^ Simmler et al. (2013), and ^d^ Rickli et al. (2015).

## Data Availability

Not applicable.
